# Virtual reality in surgery: minimizing stress and pain in patients undergoing minor-surgical procedures under local anesthesia—results of a feasibility study

**DOI:** 10.1007/s11548-024-03305-w

**Published:** 2024-12-21

**Authors:** Mine Sargut, Alexander Novotny, Helmut Friess, Michael Kranzfelder

**Affiliations:** 1 Department of Surgery, Klinikum Rechts Der Isar, TU Munich, Ismaningerstr. 22, 81675 Munich, Germany; 2 Department of Surgery, Klinikum Freising GmbH, Alois-Steinecker-Straße 18, 85354 Freising, Germany; 3 Department of Surgery, DIAKONEO KdöR, Klinik Hallerwiese-Cnopfsche Kinderklinik, Sankt-Johannis-Mühlgasse 19, 90419 Nürnberg, Germany

**Keywords:** Virtual reality (VR), VR headset, VR glasses, Port implantation, Pain, Anxiety, Stress, Feasibility study

## Abstract

**Background:**

Virtual reality (VR) technology has gained significant importance in medical practice, particularly as an innovative approach to enhance patient experience and comfort. This prospective feasibility study investigates the impact of using VR headsets on stress and pain reduction during port surgeries under local anesthesia.

**Methods:**

In this prospective analysis, patients undergoing port implantation at the Klinikum rechts der Isar Technical University Munich were divided into two groups. The intervention group wore VR headsets during the surgical procedure, while the control group did not. Various validated questionnaires were used to measure psychological parameters such as pain perception, stress, calmness, and relaxation. The amount of local anesthesia administered was also documented.

**Results:**

The study results showed that patients in the VR group required significantly less local anesthesia than those in the control group (*p* = 0.0025). Pain perception in the VR group was significantly lower (*p* = 0.028). Additionally, self-assessments regarding calmness, relaxation, and satisfaction were significantly improved in the VR group (*p* < 0.01). A stronger correlation between higher pain catastrophizing scale scores and anesthetic requirements was observed in the VR group, suggesting that VR may offer particular benefits for patients with increased pain sensitivity. Although gender differences were not statistically significant, a trend toward higher anesthetic requirements in male patients was noted. The evaluation of the VR headsets by patients was overwhelmingly positive, indicating high acceptance of the technology.

**Discussion:**

The use of VR headsets during surgical procedures can significantly enhance patient comfort by reducing pain and stress levels. The high patient acceptance and positive evaluations of VR suggest its feasibility for broader clinical application. However, future studies should address potential cognitive biases, compare VR with other distraction methods, and explore its effects on different patient subgroups. Future research should also consider the role of gender-specific factors in the modulation of anesthetic requirements by VR. Additionally, a comprehensive cost–benefit analysis will be crucial for assessing the economic viability of VR technology in healthcare.

**Conclusion:**

VR technology represents a promising method to improve patient experience and comfort in surgical settings. The positive outcomes of this study encourage further research to fully capture and validate the potential of VR in medicine, particularly in pain management and stress reduction during various medical procedures.

## Introduction

In recent years, the development of virtual reality (VR) technologies has made impressive strides, creating diverse applications across various fields. Notably, in healthcare and specifically in surgery, VR has shown transformative effects. VR glasses are increasingly being used as an innovative tool for enhancing surgical precision and for training medical professionals in real-time scenarios. This technology also offers significant benefits in the treatment of phobias, anxiety disorders, post-traumatic stress disorders, as well as in pain management and rehabilitation.

The first successful applications of VR in the medical field date back over two decades when it was used for pain treatment in burn victims [1]. Since then, evidence of VR's effectiveness as a strategy for alleviating acute pain in a variety of medical procedures for both adults and children has steadily increased [2, 3]. VR distracts attention from painful stimuli and reduces their processing in the brain by stimulating the visual cortex [4, 5]. Furthermore, the use of VR glasses has also proven effective in reducing preoperative anxiety [6].

The aim of this study is to investigate the effects of VR glasses on pain perception and stress levels during port implantation under local anesthesia.

**Fig. 1:**
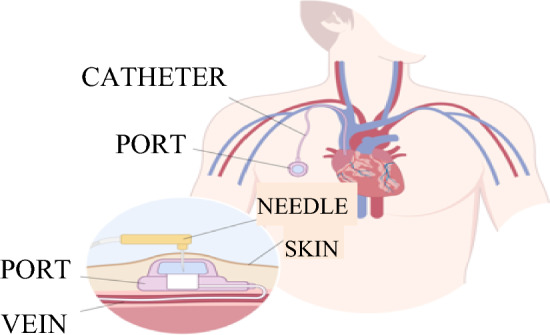
Visual description of a pectoral port system

Port surgeries are routine procedures in surgery and are frequently performed. Ports are primarily used for administering medications such as chemotherapy and parenteral nutrition and for blood sampling, mainly in cancer patients, see Fig. 1. Enhancing patients' well-being during such procedures not only directly impacts the patients themselves but also enables surgeons to work in a calmer and more efficient environment.

While, earlier VR devices tended to be expensive and non-portable, the advent of affordable devices like head-mounted displays (HMD) has made VR more practical for clinical use [7]. We hypothesize that VR devices can reduce intraoperative pain and stress in patients. Preliminary study data already revealed a positive effect of VR towards the reduction of pain and stress [8].

## Methods

This prospective analysis aimed to examine the impact of using virtual reality (VR) headsets on psychological and physiological parameters in patients. The study period ranged from February 22, 2022, to January 13, 2023. Patients were recruited from those undergoing port implantation at the Klinikum rechts der Isar, Technical University Munich, specifically at the clinic for surgery in the center for ambulatory surgery (ZAC). Inclusion criteria included a minimum age of 18 years. All Patient had no prior experience with VR headsets. All participants received general information about the study and the surgical procedures. After a comprehensive briefing and providing written consent, patients were randomized into two groups blinded. Data collection was conducted using questionnaires and tables to ensure the anonymity of patient data for privacy protection. The study was approved by the hospital's ethics committee.

**Fig. 2:**
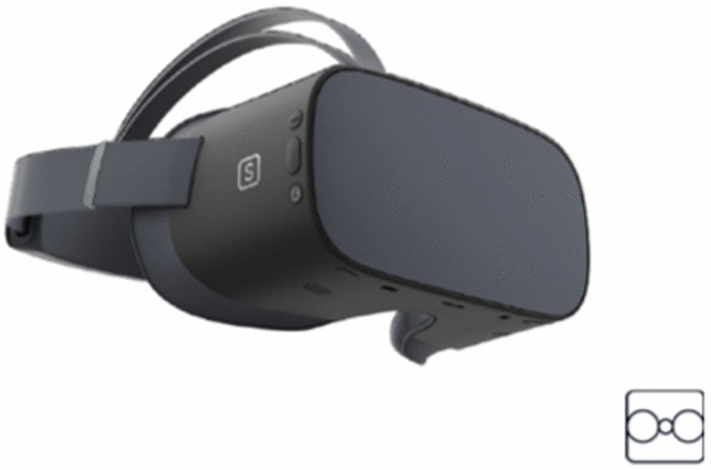
Pico G2 4 K Premium VR headsets (manufactured by SyncVR Medical GmbH, Utrecht, Netherlands)

**Fig. 3:**
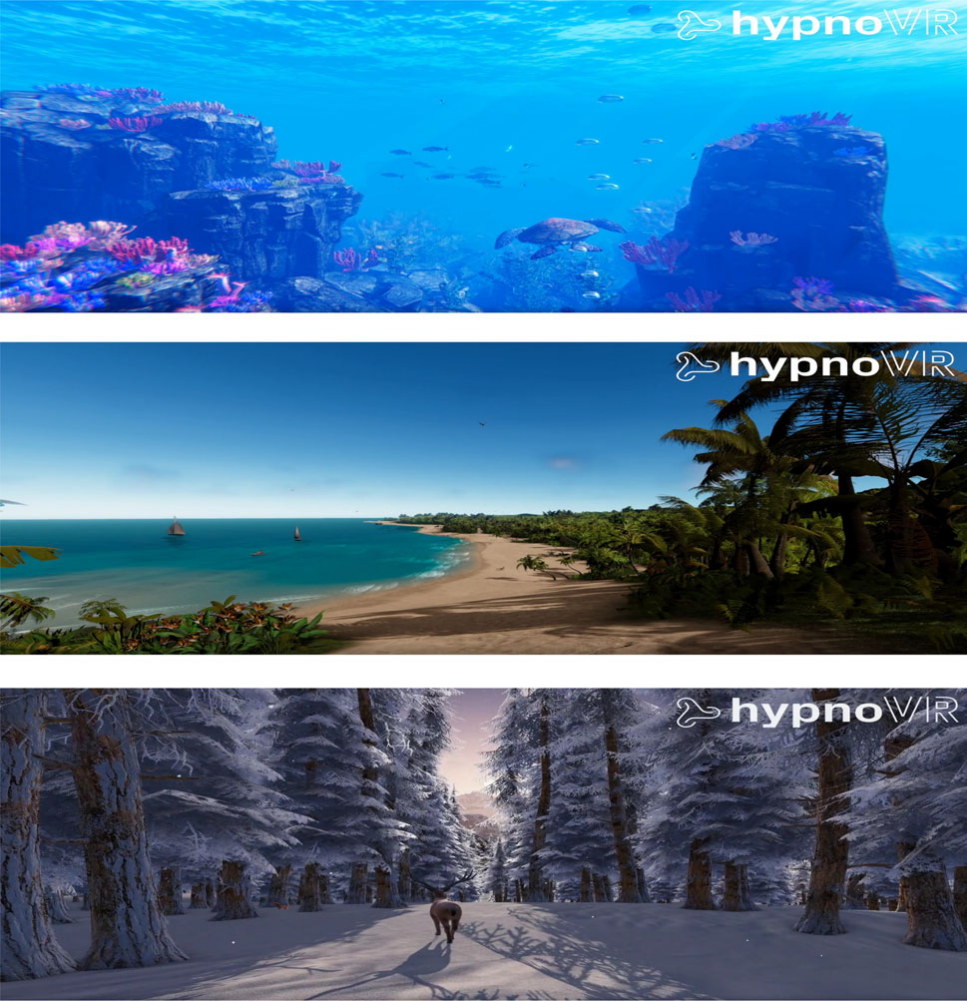
Examples of various virtual Scenarios; SyncVR Relax & Distract programs [9]

The intervention group used Pico G2 4K Premium VR headsets (manufactured by SyncVR Medical GmbH, Utrecht, Netherlands, Fig. 2) equipped with the SyncVR Relax & Distract programs [9]. Patients could choose from various virtual scenarios (e.g., underwater world, beach, winter landscape, forest walk, space, Fig. 3) and background music genres (Jazz, Lounge, Classical) projected into the VR headset. This constituted passive audiovisual VR immersion, not considered a form of medical hypnosis. The control group did not use VR glasses but had access to a music program of their choice.

Both groups underwent the surgical procedure under local anesthesia. The amount of local anesthetic administered was documented, starting with an initial dose of 20 ml of 2% Mepivacaine. Additional local anesthetic was administered as needed, in case of persistent pain during the procedure.

Various validated questionnaires were used to assess pain perception and the extent of anxiety and stress during the procedure. Additionally, demographic data such as gender and age were recorded. The study utilized several questionnaires to capture the psychological aspects in detail:

- The pain catastrophizing scale (PCS) [10] was used to assess the extent of catastrophizing thoughts in pain. The PCS comprises 13 items capturing patients' thoughts and feelings regarding pain, with a higher score indicating more intense catastrophizing. We evaluated it preoperative.
- The Y-6-Item Questionnaire [11] was used both before and after the surgery to capture patients' self-assessments regarding various emotional states. It measures six parameters: calmness, anxiety, anger, relaxation, satisfaction, and concern on a scale from 1 to 5. We evaluated it pre- and postoperative.
- The Short McGill Pain Questionnaire [12] quantified patients' pain perception. The 'McGill Total' score, consisting of various pain categories, was captured, with a higher total score indicating more intense pain perception. We evaluated it postoperative.
- The evaluation of the VR glasses by the users was conducted using a special questionnaire [9], provided by the manufacturer. It covered aspects like user-friendliness, complexity, technical assistance, features, inconsistencies, learning effort, cumbersomeness, and safety. We evaluated it also postoperative.

**Fig. 4:**
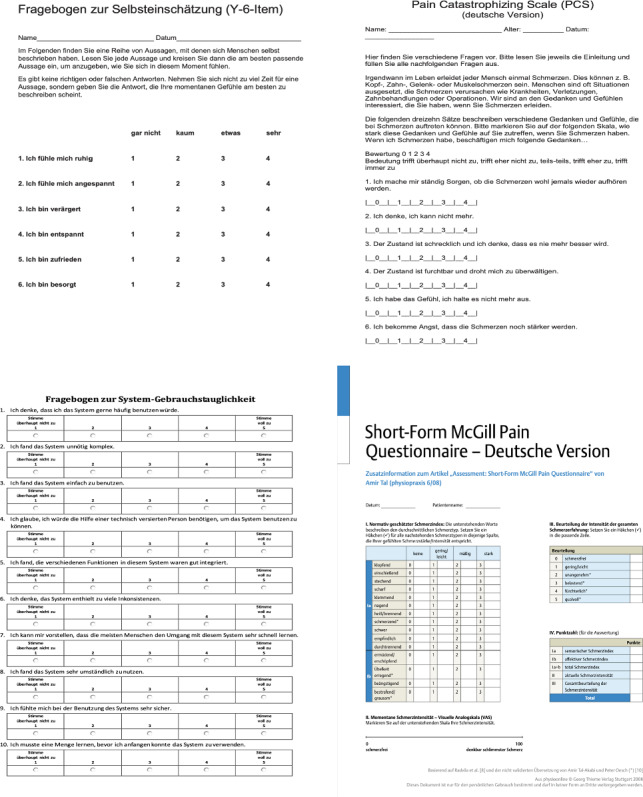
Examples of the different questionnaires used. For a detailed view, please go to the references [1–4]

The statistical analysis of the data was performed using SPSS Version 29 for Mac. The statistical analysis included independent t-tests, correlation analyses, and multiple regression analyses, with significance set at a level of *p* < 0.05. We used the Asterisk Symbol (*) to indicate the level of significance (*p < 0.05, **p < 0.01, ***p < 0.001). Data are expressed as mean ± standard deviation (SD). Summary data corresponding to each regression analysis are provided to offer insightful context. Graphs and diagrams for the visual representation of the results were also created with SPSS, to allow consistent and precise visualization of the data. Error bars in the figures represent the standard deviation (SD) of the data, indicating the variability of the individual measurements around the mean. The drafting of the study text was done in Word 2019 for Mac (Fig. 4).

## Results

A total of 60 patients were included in our study, with half (30 patients) wearing VR glasses during their surgery. The participants comprised 28 women and 32 men. In the group without VR glasses, there were 15 women and 15 men, whereas in the group with VR glasses, there were 13 women and 17 men, see Table 1.

**Table 1:** Distribution of patients by gender and use of VR headset

Patient	VR	Non-VR	Total
	n	n	
Woman	13	15	28
Men	17	15	32
Total	30	30	60

### 3.1 Pain catastrophizing scale (PCS) evaluation (10)

**Fig. 5:**
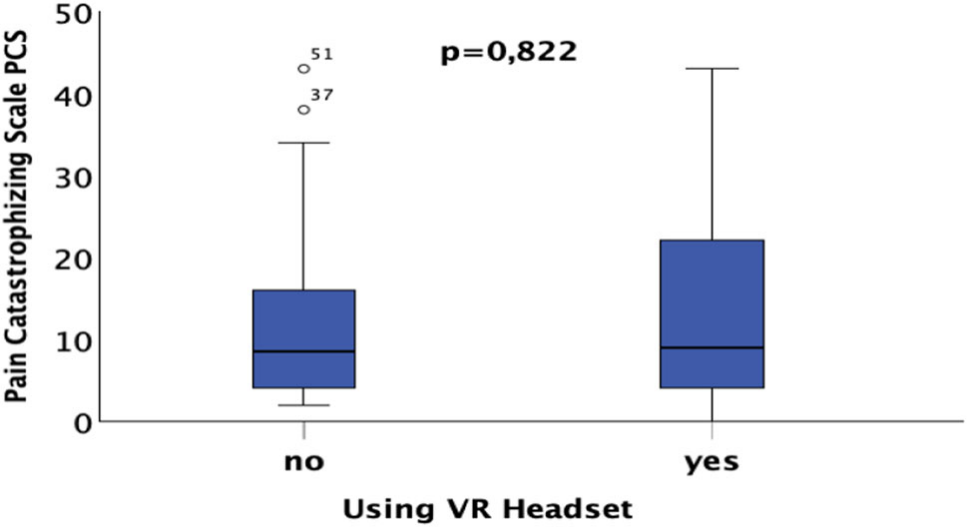
The pain catastrophizing scale (PCS) is equivalent in both patient groups, indicating that the groups have approximately the same psychological baseline

First, we analyzed the PCS and found no significant differences in catastrophizing perception between the two groups, as shown in Fig. 5. This indicates that both groups had a comparable psychological baseline, which is crucial for accurately evaluating the effects of future therapeutic interventions*.* These results emphasize the importance of a comparable psychological baseline in both patient groups for the evaluation of future therapeutic measures.

The results of the regression analysis with PCS (Pain Catastrophizing Scale) as the dependent variable and age, gender, and the use of a VR headset as independent variables are shown in Table 2.

**Table 2:** Older patients have a lower PCS score and require less local anesthesia. Male patients, however, tend to have higher PCS scores and require more local anesthesia when not using VR glasses. Higher PCS scores are associated with an increased amount of local anesthesia in both groups. The Pearson correlation between PCS scores and the amount of local anesthesia shows that the correlation is not random

Variable	PCS (p)	Local anesthesia (LA) with VR (p)	Local anesthesia (LA) without VR (p)
Age	−0.2753 (0.049)*	−0.0444 (0.177)	−0.0737 (0.485)
Gender (Male)	3.3499 (0.275)	−0.3916 (0.559)	5.5478 (0.033)*
PCS	–	0.0992 ( 0.000)***	0.2768 (0.007)**
Pearson Correlation r (PCS&LA)		0.637 (0.00015)***	0.502 (0.0047)**

In summary, age has a significant but small effect on the PCS score, while gender and the use of a VR headset show no statistically significant impact in this model.

### 3.2 Local anesthesia dosage

**Fig. 6:**
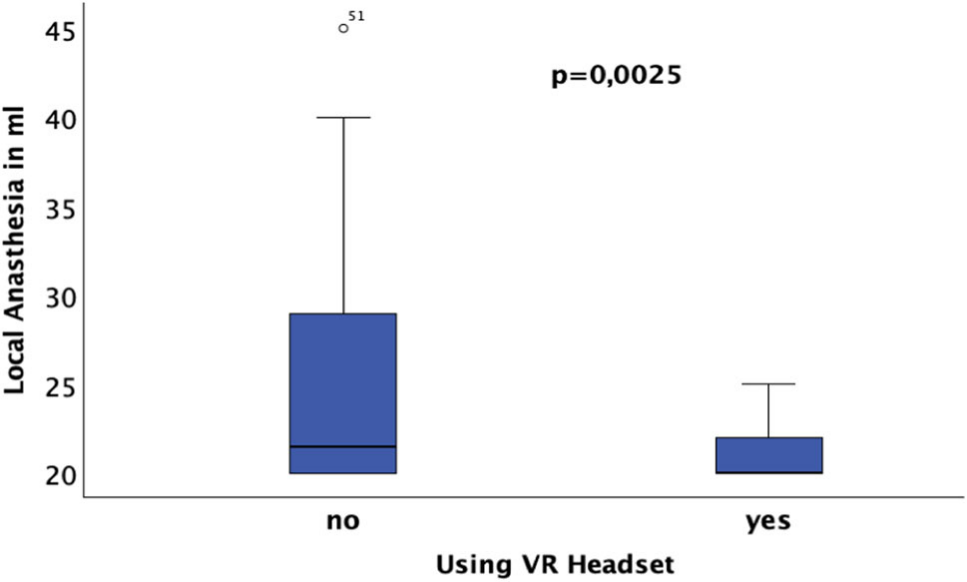
Patients using the VR headset require significantly less local anesthesia

The average amount of local anesthesia administered differed significantly between the two groups. Patients without VR headset required an average of 24.93 ml (± 6.64), while patients with VR headset received an average of 20.97 ml (± 1.77), *p* = 0.0025**, see Fig. 6. Patients using the VR headset required significantly less local anesthesia.

A gender-specific analysis revealed that male patients on average required more local anesthesia (23.84 ml ± 6.43) compared to female patients (21.93 ml ± 3.16). However, this difference was not statistically significant (*p* = 0.14).

The results of the regression analyses for the amount of local anesthesia in ml as the dependent variable in relation to age, gender, and PCS are shown in Table 2.

The PCS score has a significant impact on the amount of local anesthesia in both groups (with and without VR glasses). In the group without VR glasses, gender also has a significant impact. Men require significantly more local anesthesia than women. In the group with VR glasses, there is no difference. Based on these results, it could be asserted that men are more influenced by the glasses. Age shows no significant influence in either group.

The Pearson correlation between the PCS score and the amount of local anesthesia shows the correlation is not random. Patients with higher PCS scores tend to receive larger amounts of local anesthesia. Interestingly, the correlation is stronger in the group that used VR glasses.

### 3.3 Y-6-Item questionnaire for self-assessment (2)

The analysis of patients' self-assessments before and after surgery showed that patients with VR glasses postoperatively had a significant increase in levels of calmness and relaxation. However, there was no significant influence of VR glasses on patient satisfaction, as well as on levels of anger and concern.

**Fig. 7:**
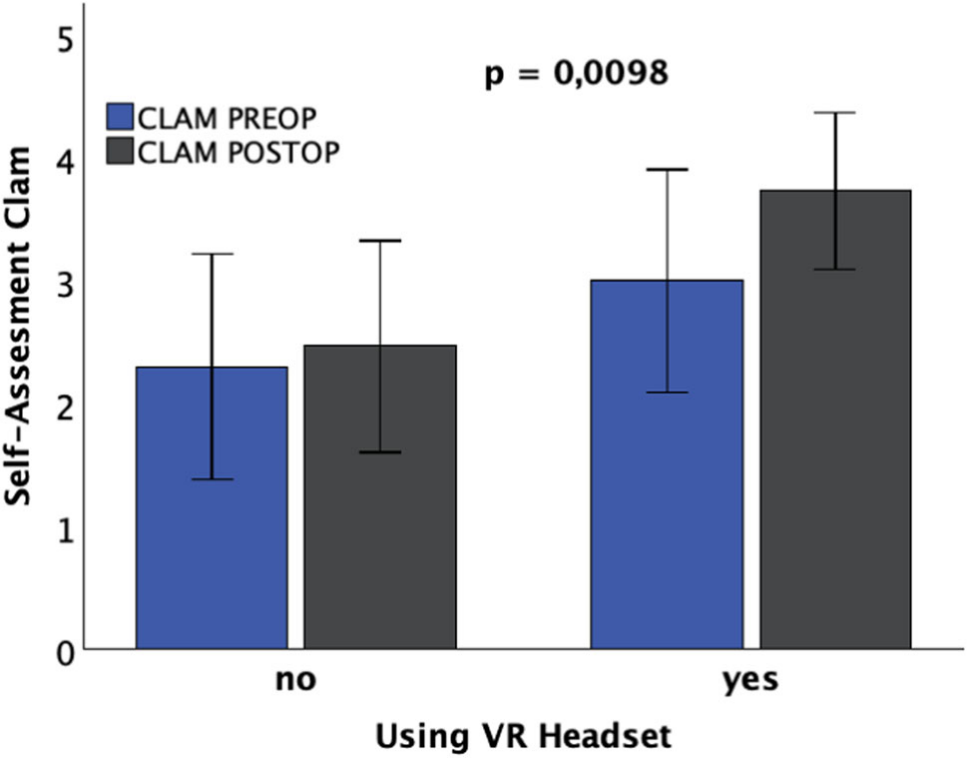
Patients with VR headset show a significant increase in calmness in their self-assessment using the Y6 questionnaire postoperatively

The improvement in the state of calmness in patients using VR glasses was statistically significant compared to patients without VR glasses (*p* = 0.0098**), see Fig. 7.

**Fig. 8:**
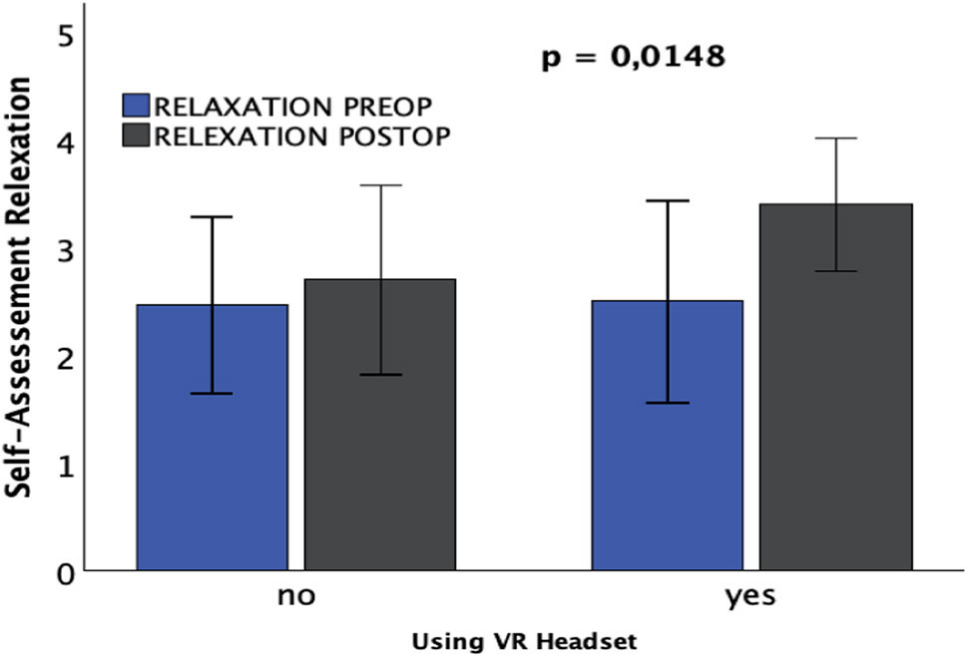
Patients with VR glasses show a significant increase in relaxation in their self-assessment using the Y6 questionnaire postoperatively

Similarly, there was a significant improvement in the level of relaxation among patients using VR glasses (*p* = 0.0148*), suggesting that the use of VR glasses effectively contributed to increasing patient relaxation after the procedure, see Fig. 8.

### 3.4 McGill total pain score (12)

**Fig. 9:**
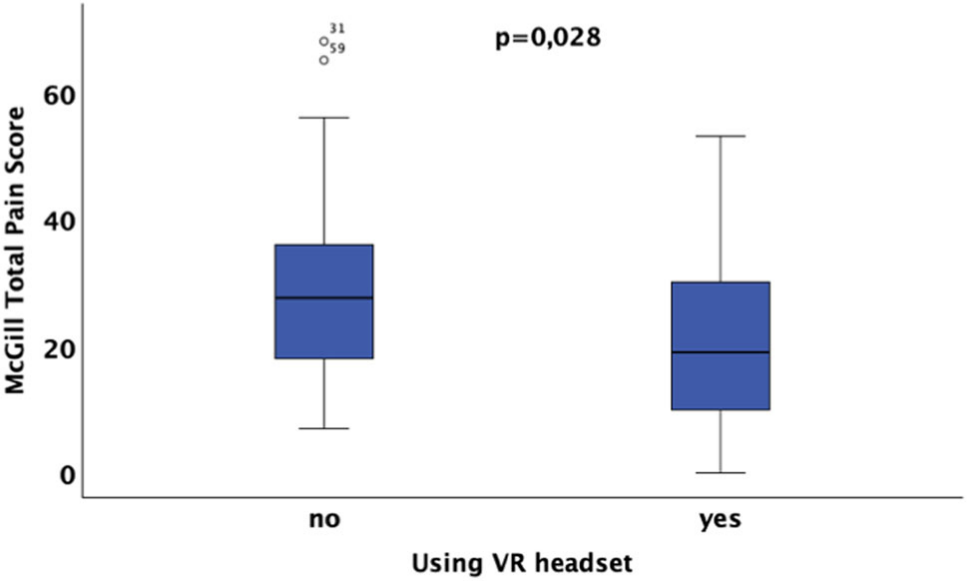
Postoperative McGill total pain scores for pain perception are significantly lower in patients with VR headset

A significant difference in the McGill total pain score for pain perception was observed between patients who used a VR headset and those who did not (*p* = 0.028*). This also suggests that the use of VR technology could have a significant impact on patients' pain perception, see Fig. 9. The regression analyses for the McGill Total Scores regarding age and gender suggest that neither age nor gender has a significant influence on the McGill Total Score of patients, regardless of whether they use a VR headset or not.

### 3.5 Evaluation of the VR headset

The study also assessed patients' perceptions of using a VR headset using a specialized questionnaire [9]. Patients rated various statements regarding the use of VR glasses on a scale from 1 (low agreement) to 5 (high agreement).

The overall feedback was very positive. Most patients expressed a strong willingness to use the VR headset regularly. Patients found the VR system to be simple and not complicated. The system was considered very user-friendly and easy to understand. Most patients were able to operate the VR system independently, needing little assistance. Patients felt that the various features of the VR system were well integrated. The use of the VR headset was perceived as safe.

**Fig. 10:**
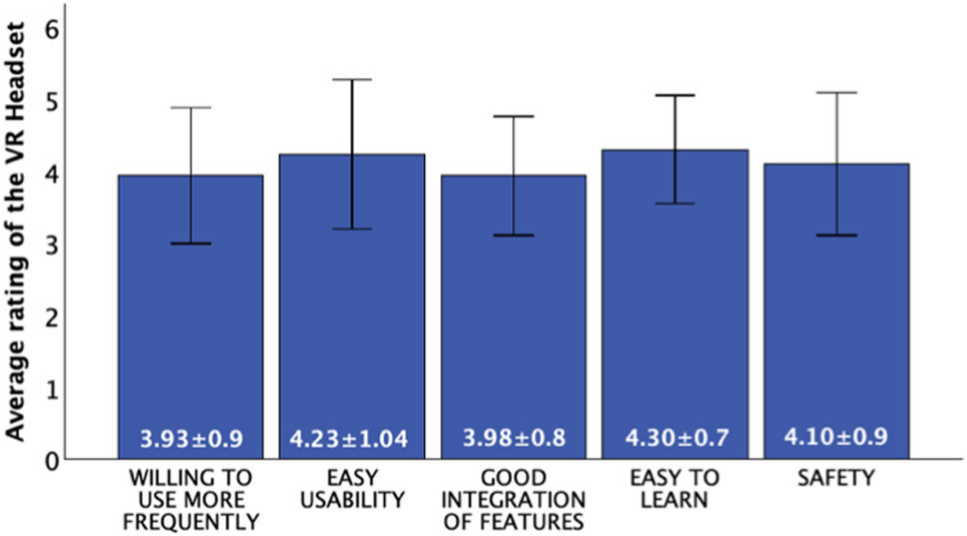
Patients perceived the use of the VR headset as functional, easy to use, easy to learn, and safe. They would like to use the VR headset more frequently

**Fig. 11:**
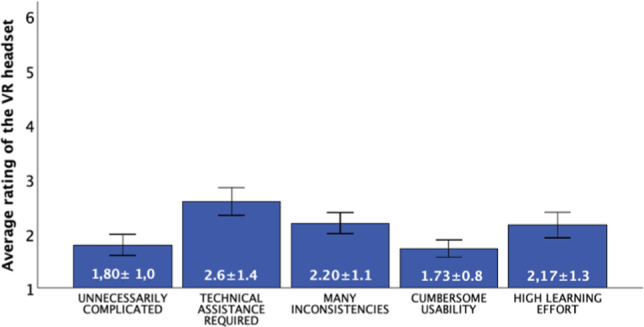
Patients found the system to be uncomplicated and believe they require little technical support. They did not perceive the usage as cumbersome and observed minimal inconsistencies. They do not believe much prelearning is necessary before use

The VR system was viewed as reliable and consistent in its performance. The system was described as easy to handle and not cumbersome. Patients found the VR system easy to learn and quickly grasped how to use it. Overall, the VR headset was described by patients as user-friendly, safe, and intuitive. These positive evaluations suggest that VR technology could be widely accepted and further implemented in healthcare settings, see Figs. 10 and 11.

## Discussion

The findings of our study suggest that the use of virtual reality (VR) headsets during surgical procedures, such as port implantations under local anesthesia, can significantly reduce the required amount of local anesthetic. The observed improvements in pain perception and increased patient well-being, as indicated by the McGill Pain Questionnaire [12] and Y-6 Item Questionnaire [11], are noteworthy. These results align with previous studies demonstrating that immersive VR can effectively modulate pain perception by providing a distraction and altering the patient's sensory experience [13]. This highlights the future potential of VR in medicine, more critical. For surgeries requiring larger amounts of local anesthetics, such as orthopedic or extensive dental procedures, the associated side effects and risks can be significant. Using VR technologies might help reduce the necessary dosage, thereby decreasing the risks of side effects, such as systemic toxicity, allergic reactions, or other complications [14].

Furthermore, our findings may be extrapolated to more complex surgical procedures, where pain management poses a challenge. VR technology could be utilized as a complementary method for pain relief, potentially reducing the required anesthetics and associated risks. The observed positive correlation between PCS (Pain Catastrophizing Scale) scores and the amount of local anesthesia used in our study suggests that VR technologies could be particularly beneficial for patients with higher pain sensitivity. Higher PCS scores have been consistently associated with increased pain intensity and greater analgesic requirements in various medical contexts [1]. However, this correlation does not establish causation, and it remains uncertain whether the analgesic effect of VR is truly independent of the anesthesia dosage required. A more refined study design, including a larger sample size and additional controls for confounding factors, would be necessary to draw definitive conclusions regarding this relationship.

### 4.1 Consideration of sex-specific factors

The gender-specific analysis revealed that male patients generally required more local anesthesia than female patients, although this difference was not statistically significant. These findings are consistent with previous studies that have documented sex-based differences in anesthesia requirements. Hormonal factors, such as the influence of testosterone, can contribute to reduced sensitivity to certain anesthetics in men, resulting in a higher dosage requirement [15]. Additionally, variations in body composition and metabolism, such as muscle mass and fat distribution, can affect the distribution and efficacy of anesthetics, leading to higher requirements for men to achieve comparable anesthetic effects [15]. These insights underscore the importance of considering sex-specific factors in anesthesia protocols to develop personalized and effective pain management strategies. Future research should aim to better understand these underlying mechanisms and optimize clinical practices accordingly.

### 4.2 Correlation between PCS scores and VR effects

While, the correlation between higher PCS scores and the amount of local anesthesia required was significant for both groups, it was stronger in the VR group. This suggests that patients with a higher tendency towards pain catastrophizing may benefit more from VR interventions. One possible explanation is that VR may help modulate the emotional and cognitive aspects of pain more effectively in individuals who experience higher levels of psychological distress associated with pain [15]. This hypothesis is supported by research showing that distraction techniques, such as VR, are particularly effective in patients who are more prone to anxiety or pain catastrophizing [10]. Future studies should explore the mechanisms behind this enhanced effect and determine how VR can be tailored to maximize its benefits for different patient subgroups.

### 4.3 Limitations and future research directions

One major limitation is the potential for cognitive biases, such as the observer-expectancy effect, the Hawthorne effect, and the halo effect, which may have influenced the subjective reports of patients. To mitigate these biases, future studies should ensure that both the treatment and control groups wear VR headsets, with the control group experiencing neutral visuals to isolate the specific effects of the VR content. Additionally, further research should compare VR with other distraction techniques, such as listening to music or watching videos, to evaluate whether VR provides unique benefits. Studies have shown that different types of distraction can have varying effects on pain perception, suggesting that the type of distraction used may need to be tailored to individual patient characteristics [16].

## Conclusion

This study supports the use of virtual reality (VR) technology as an effective tool for reducing pain and stress during surgical procedures, such as port implantations under local anesthesia. The findings indicate that VR can significantly decrease the amount of local anesthetic required, potentially reducing the risk of side effects associated with higher doses of anesthesia. Moreover, the observed correlation between higher pain catastrophizing scale (PCS) scores and anesthesia requirements suggests that VR may offer particular benefits for patients with increased pain sensitivity, though further research is needed to confirm these effects.

The gender-specific analysis, while not statistically significant, indicated a trend toward higher anesthesia requirements in male patients. This finding highlights the importance of considering sex-specific factors, such as hormonal influences and body composition, in developing personalized pain management strategies. Future studies should aim to better understand these differences and their implications for clinical practice.

Although the results are promising, this study has limitations, including the potential for cognitive biases and the need for more rigorous control of confounding variables. Further research with larger sample sizes and well-designed control conditions is necessary to validate these findings and establish the unique benefits of VR compared to other distraction techniques.

In conclusion, VR represents a promising adjunct to conventional pain management methods in surgical settings. Continued exploration of its mechanisms and optimal use cases will be crucial to fully realize its potential in clinical practice.
